# The dawn of stomatal control by the hormone of darkness

**DOI:** 10.1093/jxb/erag243

**Published:** 2026-07-29

**Authors:** Daniela Aros-Mualin, Scott A M McAdam

**Affiliations:** Department of Botany and Plant Pathology, Purdue University, West Lafayette, IN 47907, USA; Department of Botany and Plant Pathology, Purdue University, West Lafayette, IN 47907, USA

**Keywords:** Abscisic acid, angiosperms, ferns, gymnosperms, lycophytes, melatonin, salicylic acid, SLAC1

## Abstract

This article comments on:

**Liu X-D, Ren Y-L, Li Y-L, Zeng Y-Y, Zhang X-Y, Li Y-R, Du Y-X, Fang X-W**. 2026. Lineage-specific response to melatonin in stomatal regulation across vascular plants. Journal of Experimental Botany **77**, 4668–4678. https://doi.org/10.1093/jxb/erag146

This article comments on:

**Liu X-D, Ren Y-L, Li Y-L, Zeng Y-Y, Zhang X-Y, Li Y-R, Du Y-X, Fang X-W**. 2026. Lineage-specific response to melatonin in stomatal regulation across vascular plants. Journal of Experimental Botany **77**, 4668–4678. https://doi.org/10.1093/jxb/erag146

**Stomata, the microscopic pores on leaf surfaces that regulate gas exchange, are known to respond differently to hormonal signals among vascular land plant lineages. In a new study, Liu *et al*. (2026) add a critical piece of evidence to this growing body of literature, showing that melatonin, a hormone that regulates day–night cycles across kingdoms of life, drives nocturnal stomatal closure in seed plants but not in ferns or lycophytes. Here we explore what this lineage-specific response reveals about the evolution of stomatal regulation in vascular plants.**

The daytime–night-time cycling of stomatal opening to allow photosynthesis in the light and closure in the dark is an ancestral response of vascular land plants (Ivisic *et al*., 2025). Work in Arabidopsis has found that the hormone melatonin, whose function has long been described in regulating day–night cycles in mammals, also regulates day–night stomatal cycles (Wei *et al*., 2018; Li *et al*., 2020). In exciting new work, Liu *et al*. (2026) show that melatonin regulation of stomatal day–night cycles is unique to seed plants. This discovery adds to a considerable body of literature suggesting that stomata of seed plants are highly sensitive to metabolic regulators, whereas the stomata of seed-free plants are unresponsive to hormones (Brodribb and McAdam, 2011, 2013; Westbrook and McAdam, 2020b; Gong *et al*., 2021; Aros-Mualin *et al*., 2023; Sussmilch *et al*., 2024; Li *et al*., 2025; Ivsic *et al*., 2025; Zeng *et al*. 2025; Zhang *et al*., 2025).

The results of Liu *et al*. (2026) are particularly interesting because they reveal a series of melatonin-induced signalling patterns in guard cells with varying degrees of conservation across vascular plants. In all vascular plants, the application of low concentrations (<10 μM) of melatonin to guard cells induces an accumulation of reactive oxygen species (ROS). This indicates that all plants can detect hormonal levels of melatonin. After this initial response, there is considerable divergence between plants with seeds and those without. In seed plants, nitric oxide (NO) and subsequently [Ca^2+^] increase in the guard cells, and stomata close. In seed-free plants, there is no such response of the guard cells, and stomata do not close (Fig. 1). Liu *et al*. (2026) conclude that melatonin plays a vital role in the circadian regulation of stomata not only in Arabidopsis (Li *et al*., 2020) but in all seed plants. The absence of this response in seed-free plants aligns with recent work showing that most seed-free plants do not have a circadian regulation of stomata (Aros-Mualin *et al*., 2023). In seed plants, the day–night cycling of stomatal aperture is controlled not only by light but also by a periodic 24 h intrinsic rhythm, which results in opening and closing cycles even when exposed to constant light or darkness (Hassidim *et al*., 2017; Simon *et al*., 2020). In Arabidopsis, the oscillator that regulates the whole-plant internal circadian clock differs from the guard cell oscillator. This means that the internal self-sustained molecular mechanism driving circadian cycles in guard cells differs from that of the whole plant (Hassidim *et al*., 2017), suggesting an independent evolution. This supports the conclusions of Aros-Mualin *et al*. (2023) and Liu *et al*. (2026) that circadian regulation of stomata evolved in a common ancestor of seed plants by localizing and modifying the already existent whole-plant internal circadian clock in guard cells.

**Fig. 1.**
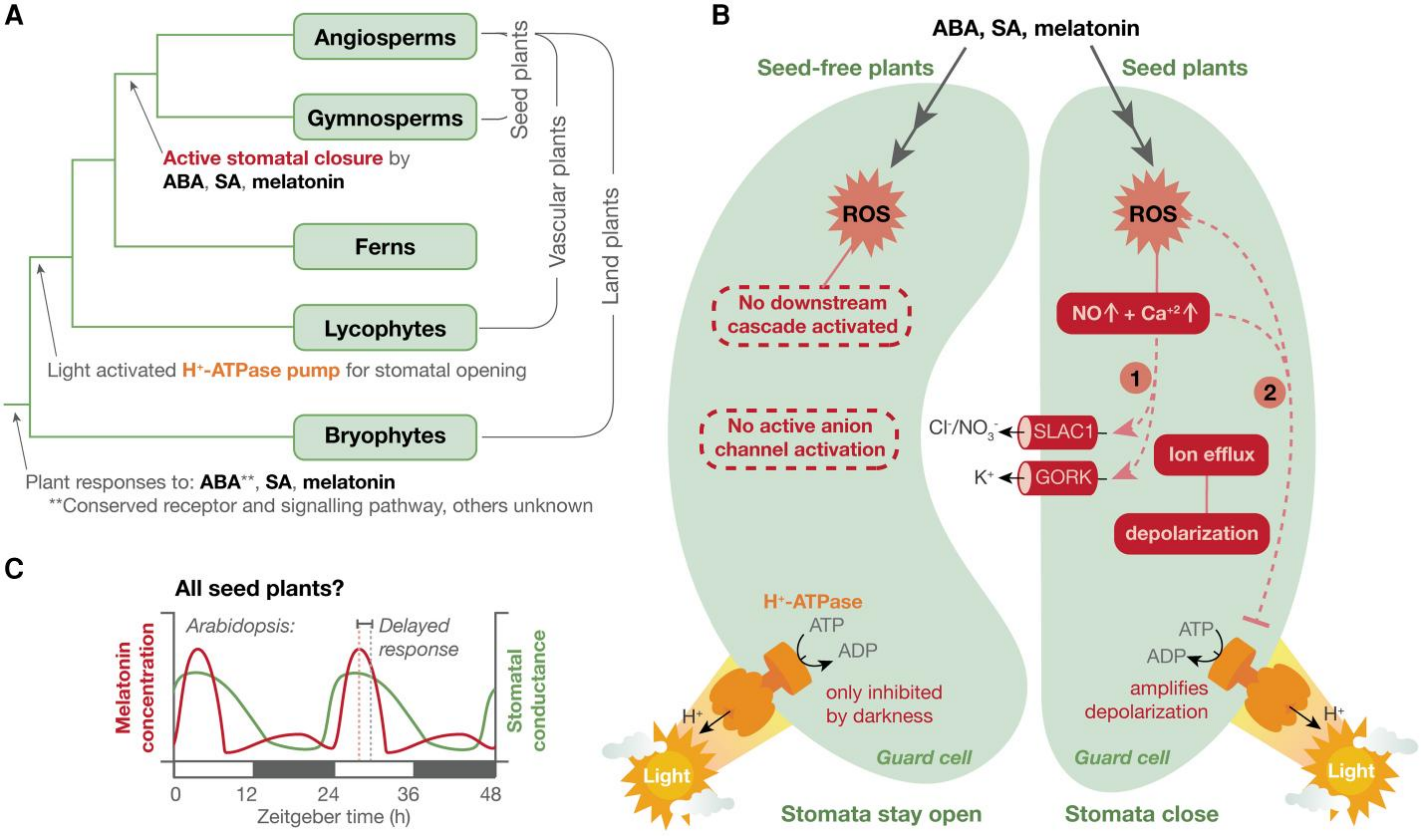
Evolution of active stomatal closure by hormones in seed plants. (A) Phylogeny of vascular land plants showing two distinct evolutionary origins of stomatal responses to hormones: an ancestral origin of cellular perception of abscisic acid (ABA), salicylic acid (SA), and melatonin across all vascular plant lineages, and the later evolution of hormone-triggered stomatal closure restricted to seed plants. (B) Stomatal responses to ABA, SA, and melatonin in seed-free vascular plants (ferns and lycophytes) and seed plants. All vascular plant lineages open stomata in response to light and close in darkness, but only seed plants close stomata in the light through a hormone-triggered signalling cascade that activates outward-rectifying S-type anion channels (SLAC1) and outward-rectifying K^+^ channels (GORK) in the guard cell plasma membrane. Despite lacking this downstream cascade, guard cells of seed-free vascular plants detect all three hormones, as evidenced by a ROS burst (primarily H_2_O_2_) inside the guard cell. Dotted lines indicate inferred steps lacking direct experimental evidence, including: (1) melatonin-induced stomatal closure acting through SLAC1 activation, and (2) increases in ROS, NO, or Ca^2+^ inhibiting the plasma membrane H ^+^ -ATPase. (C) Diel patterns of melatonin concentration and stomatal conductance over two light/dark cycles in *Arabidopsis thaliana* (adapted from Li *et al*., 2020). Whether equivalent diel patterns occur in gymnosperms or other angiosperm lineages remains unknown.

Species from the family *Marsileaceae* constitute an exception to an absence of circadian regulation of stomata in seed-free plants (Aros-Mualin *et al*., 2022, 2023). This unique, angiosperm-like, amphibious group of ferns has circadian regulation of stomata; however, as shown by Liu *et al*. (2026), these responses are not controlled by melatonin. As with stomatal circadian rhythms, *Marsileaceae* have also independently evolved an angiosperm-like stomatal opening by lateral displacement of guard cells into neighbouring epidermal cells, and stomatal responses to low fluence blue light (Westbrook and McAdam, 2020a, b). The finding by Liu *et al*. (2026) that circadian regulation of stomata in *Marsileaceae* does not require melatonin has a number of important implications. Firstly, it suggests that the lack of circadian regulation of stomata in seed-free plants is unlikely to be the result of a loss of function across seed-free lineages, but rather the independent evolution of this response through different pathways in seed plants and *Marsileaceae*. Secondly, it shows that circadian rhythms can be regulated by other processes in addition to melatonin. We do not know yet what these mechanisms might be, but one hypothesis is that circadian rhythms of stomata in *Marsileaceae* are passive responses to changes in leaf water status. In support of this, leaf nyctinasty is also under circadian control in *Marsileaceae* and, given that these responses are driven by localized changes in cell turgor in the pulvinus, changes in leaf water status could provide a common mechanism for controlling both processes simultaneously (Aros-Mualin *et al*., 2022).

The fundamental differences in stomatal regulation by melatonin across vascular plant lineages mirrors work in a range of other hormones and signalling molecules. Seed-free plants, including *Marsileaceae*, have stomata that are insensitive to the hormone abscisic acid (Brodribb and McAdam, 2011; Westbrook and McAdam, 2020b; Gong *et al*., 2021; Li *et al*., 2025), salicylic acid (Zhang *et al*., 2025), and other signals (Brodribb and McAdam, 2013; Zeng *et al*. 2025). A shared feature of many of these metabolic drivers of stomatal closure is that they converge on the activation of outward-rectifying anion channels in the guard cell plasma membrane to promote stomatal closure (Prodhan *et al*., 2018; Sussmilch *et al*., 2024). Work in model ferns and lycophytes suggests that there is an absence of outward-rectifying anion channels that can be activated in the guard cells of these species (Sussmilch *et al*., 2024). We do not know yet if the melatonin signalling pathway triggers the activation of guard cell outward-rectifying anion channels, such as SLAC1, in seed plants. In any case, the work of Liu *et al*. (2026), as well as many other studies, indicates that the minimal metabolic control of stomata in seed-free plants reflects not only the absence (or loss) of activatable outward-rectifying anion channels in guard cells, but also the absence of the upstream guard cell signalling cascade that would activate them (Gong *et al*., 2021).

All hormones that play a critical role in stomatal regulation in seed plants have important functions outside guard cells across land plants. For example, abscisic acid regulates sex determination, spore and seed dormancy, and general stress responses, while melatonin regulates processes from seed germination, to root and shoot growth, secondary metabolite production, and protection of photosynthesis under stress (McAdam *et al*., 2016; Arnao and Hernández-Ruiz, 2019; Yang *et al*., 2022). These non-stomatal functions of stomata-regulating hormones probably indicate an ancestral role for these hormones in plants. Previous work suggests that melatonin may regulate photosynthetic protection under stress in algae, particularly under high light conditions, which might have been critical for terrestrialization (Zhao *et al*., 2021; Yang *et al*., 2022). We do not yet know if melatonin regulates any other day–night cycling outside seed plants.

A major question arising from the work of Aros-Mualin *et al*. (2023) and Liu *et al*. (2026) is: why did the seed plants evolve circadian regulation of stomata? Closure of stomata in the dark in non-seed plants is extremely slow compared with seed plants (Kübarsepp *et al*., 2020). It could be that the presence of circadian regulation increases the speed of stomatal closure in the dark (Li *et al*., 2020). Circadian regulation of stomata might also prime necessary metabolic pathways for stomatal opening before the dark-to-light transition in the morning, promoting more rapid stomatal opening, thereby minimizing stomatal limitation to photosynthesis in the morning. Along these lines, it might be that the considerable metabolic machinery that evolved to actively close stomata in seed plants requires some degree of deactivation to allow stomata to open sufficiently quickly in the morning. Fern stomata, without the burden of considerable metabolic pathways driving stomatal closure, open very rapidly in the light (Brodribb and McAdam, 2013; Kübarsepp *et al*., 2020). Circadian enhancement of stomatal response time during light transitions in seed plants could both enhance stomatal control and improve water use efficiency compared with seed-free plants (Simon *et al*., 2020), which may have contributed to the global ecological dominance of this group.
